# African Swine Fever Virus Armenia/07 Virulent Strain Controls Interferon Beta Production through the cGAS-STING Pathway

**DOI:** 10.1128/JVI.02298-18

**Published:** 2019-05-29

**Authors:** Raquel García-Belmonte, Daniel Pérez-Núñez, Marco Pittau, Juergen A. Richt, Yolanda Revilla

**Affiliations:** a Centro de Biología Molecular Severo Ochoa, CSIC-UAM, Universidad Autónoma de Madrid, Madrid, Spain; b Università degli Studi di Sassari, UNISS, Dipartimento di Medicina Veterinaria, Sassari, Italy; c Department of Diagnostic Medicine/Pathobiology, College of Veterinary Medicine, Kansas State University, Manhattan, Kansas, USA; University of Illinois at Urbana Champaign

**Keywords:** ASFV, Armenia/07, IFN-β, NH/P68, STING, cGAMP, cGAS, virulence

## Abstract

African swine fever virus (ASFV) is a complex, cytoplasmic double-stranded DNA (dsDNA) virus that is currently expanding throughout the world. Currently, circulating virulent genotype II Armenia/07-like viruses cause fatal disease in pigs and wild boar, whereas attenuated strains induce infections with various levels of chronic illness. Sensing cytosolic dsDNA, mainly by the key DNA sensor cyclic GMP-AMP synthase (cGAS), leads to the synthesis of type I interferon and involves signaling through STING, TBK1, and IRF3. After phosphorylation, STING translocates from the endoplasmic reticulum to the Golgi compartment and to the perinuclear region, acting as an indispensable adaptor connecting the cytosolic detection of DNA to the TBK1-IRF3 signaling pathway. We demonstrate here that attenuated NH/P68, but not virulent Armenia/07, activates the cGAS-STING-IRF3 cascade very early during infection, inducing STING phosphorylation and trafficking through a mechanism involving cGAMP. Both TBK1 and IRF3 are subsequently activated and, in response to this, a high level of beta interferon (IFN-β) was produced during NH/P68 infection; in contrast, Armenia/07 infection generated IFN-β levels below those of uninfected cells. Our results show that virulent Armenia/07 ASFV controls the cGAS-STING pathway, but these mechanisms are not at play when porcine macrophages are infected with attenuated NH/P68 ASFV. These findings show for the first time the involvement of the cGAS-STING-IRF3 route in ASFV infection, where IFN-β production or inhibition was found after infection by attenuated or virulent ASFV strains, respectively, thus reinforcing the idea that ASFV virulence versus attenuation may be a phenomenon grounded in ASFV-mediated innate immune modulation where the cGAS-STING pathway might play an important role.

**IMPORTANCE** African swine fever, a devastating disease for domestic pigs and wild boar, is currently spreading in Europe, Russia, and China, becoming a global threat with huge economic and ecological consequences. One interesting aspect of ASFV biology is the molecular mechanism leading to high virulence of some strains compared to more attenuated strains, which produce subclinical infections. In this work, we show that the presently circulating virulent Armenia/07 virus blocks the synthesis of IFN-β, a key mediator between the innate and adaptive immune response. Armenia/07 inhibits the cGAS-STING pathway by impairing STING activation during infection. In contrast, the cGAS-STING pathway is efficiently activated during NH/P68 attenuated strain infection, leading to the production of large amounts of IFN-β. Our results show for the first time the relationship between the cGAS-STING pathway and ASFV virulence, contributing to uncover the molecular mechanisms of ASFV virulence and to the rational development of ASFV vaccines.

## Abstract (precis)

African swine fever, a devastating disease for domestic pigs and wild boar, is currently spreading in Europe, Russia, and China, becoming a global threat with huge economic and ecological consequences. One interesting aspect of ASFV biology is the molecular mechanism leading to high virulence of some strains compared to more attenuated strains, which produce subclinical infections. In this work, we show that the presently circulating virulent Armenia/07 virus blocks the synthesis of IFN-β, a key mediator between the innate and adaptive immune response. Armenia/07 inhibits the cGAS-STING pathway by impairing STING activation during infection. In contrast, the cGAS-STING pathway is efficiently activated during NH/P68 attenuated strain infection, leading to the production of large amounts of IFN-β. Our results show for the first time the relationship between the cGAS-STING pathway and ASFV virulence, contributing to uncover the molecular mechanisms of ASFV virulence and to the rational development of ASFV vaccines.

## INTRODUCTION

Pathogen associated molecular patterns (PAMPs) are recognized by pattern recognition receptors, which activate signal transduction pathways in order to induce innate immune responses such as type I interferon (IFN) production. Cytoplasmic double-stranded DNA (dsDNA) acts like a potent PAMP, sensed by cyclic GMP-AMP synthase (cGAS). cGAS detects cytoplasmic dsDNA and catalyzes the synthesis of GMP-AMP cyclic dinucleotide (cGAMP) (1, 2). cGAMP acts as a second messenger and binds to the stimulator of interferon genes protein (STING), which traffics from the endoplasmic reticulum (ER) to the *trans*-Golgi network (TGN), where TANK-binding kinase 1 (TBK1) is recruited and phosphorylated. This event allows the recruitment of IRF3 that is subsequently phosphorylated by TBK1 and then translocates to the nucleus, where it acts as a transcription factor for type I IFN-β gene expression (2–4).

African swine fever virus (ASFV) causes a lethal disease, which poses important economic consequences to the pig industry and to the ecosystems in affected countries (5). Because there is no suitable vaccine, at present, the spread of ASFV is uncontrolled. ASFV expansion started in 2007 in the Republic of Georgia, then reached Russia, Eastern and Central Europe, and recently China (6), where more than 75 outbreaks have been declared so far. ASFV, the only member of the *Asfaviridae* family (7), is an enveloped, cytoplasmic dsDNA virus that encodes more than 150 proteins in infected macrophages, the natural target cell population (8), including proteins that have various roles in virus-host interactions and in the modulation of the immune response (9–17). However, the function of many viral gene products remains unknown (18).

In Africa, wild suidae, such as warthogs and bush pigs, are also infected with ASFV; however, they show only subclinical infections and can act as virus carriers. In contrast, acute ASF in domestic pigs or the European wild boar is characterized by hemorrhages in lymph nodes and internal organs and high temperatures, resulting in the death of the animal in about 7 to 10 days. Different strains of the virus exhibit different virulence, ranging from peracute to acute to subclinical and chronic forms of the disease (reviewed in reference 19). The fact that ASFV strains display different virulence patterns, suggests a distinctive activation of the immune system (reviewed in reference 20), resulting in a complex scenario of virus-host interactions (21–23) and type I IFN cascade (24).

Our studies show, for the first time, that virulent ASFV Armenia/07 strain has acquired specific mechanisms to control IFN-β production during infection of porcine alveolar macrophages. These mechanisms involve the inhibition of (i) cGAS-dependent viral DNA sensing, (ii) cGAMP-mediated phosphorylation of STING, (iii) STING trafficking, and (iv) TBK1/IRF3 activation. The inhibition and control of IFN-β synthesis, one of the most important antiviral immune factors, is most likely an essential feature for the virulent ASFV Armenia/07 strain. On the other hand, the induction of IFN-β by NH/P68 could further explain its *in vivo* attenuation.

## RESULTS

### Virulent ASFV Armenia/07 infection inhibits mRNA production and secretion of IFN-β.

**FIG 1:**
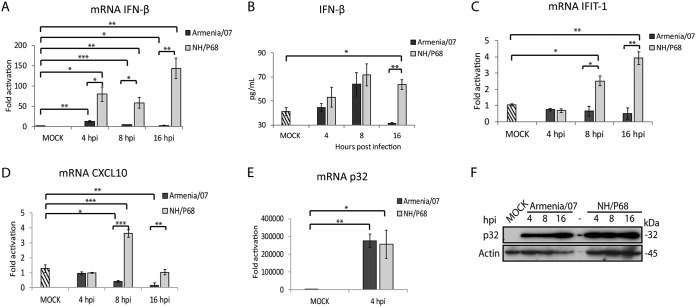
IFN-β, IFIT-1, and CXCL10 expression in porcine alveolar macrophages infected by ASFV NH/P68 or Armenia/07 strains. Porcine alveolar macrophages were mock infected or infected with NH/P68 or Armenia/07 strain (4 PFU/cell). Cells were collected at 4, 8, or 16 hpi. qPCR analyses of IFN-β (A), IFIT-1 (C), and CXCL10 (D) and of ASFV viral protein p32 (E) mRNAs were performed. (B) Supernatants were collected at 4, 8, or 16 hpi, and the concentration of IFN-β was determined by ELISA. (F) Cells were lysed in RIPA buffer at 4, 8, and 16 hpi, and lysates were separated by 7 to 20% SDS-PAGE, followed by immunoblotting with anti-ASFV p32 (viral early p32) and anti-actin antibodies. The data are means ± the standard errors of the mean (SEM; *n* = 3). Data were statistically analyzed by using a Student *t* test (*, *P* < 0.05; **, *P* < 0.01; ***, *P* < 0.001).

ASFV strains can either cause chronic, subclinical, or fatal, acute ASF disease. In order to study whether differences in ASFV virulence are related to differences in the activation of the innate immune response, we analyzed the level of IFN-β produced by porcine alveolar macrophages infected either with NH/P68 (attenuated) or with Armenia/07 (virulent) ASFV strains. For this purpose, a time course experiment in macrophages at 0, 4, 8, and 16 h postinfection (hpi) was performed. Figure 1A shows a higher production of IFN-β mRNA in cells infected with NH/P68 compared to those infected with Armenia/07, starting at 4 hpi with a maximum at 16 hpi, a time point where IFN-β mRNA was very low in cells infected with Armenia/07. Interestingly, we observed a significant increase of IFN-β mRNA in cells infected with NH/P68 from 4 to 16 hpi, indicating that cellular signaling leading to IFN-β transcription is activated during the course of the infection with the attenuated virus. Next, the amount of IFN-β secreted during attenuated versus virulent infections was determined. Supernatants from either NH/P68- or Armenia/07-infected macrophages were collected, and IFN-β levels were measured by enzyme-linked immunosorbent assay (ELISA) (Fig. 1B). The results show a strong decrease of secreted IFN-β at 16 hpi, after an initial increase at 8 hpi, during the infection with the virulent strain, whereas in NH/P68-infected macrophages the amount of IFN-β increases over time. These data correlate well with the results obtained regarding IFN-β mRNA production (Fig. 1A). This indicates that virulent ASFV shuts down the IFN-β synthesis pathway several hours after infection (Fig. 1A), although IFN-β mRNA production is significantly above the control level at 4 hpi. This effect also translates to IFN-β cytokine production, which initially increases and is totally abolished at 16 hpi, when macrophages are infected with virulent ASFV (Fig. 1B).

In addition, we analyzed the expression of IFIT-1 and CXCL10, two IFN-β-dependent genes (25, 26) at 0, 4, 8, and 16 hpi. Fig. 1C and D, show that Armenia/07 does not activate either IFIT-1 or CXCL10 mRNA synthesis during the course of the infection, whereas, in contrast, IFIT-1 and CXCL10 mRNA level increases during NH/P68 infection. Finally, and as shown in Fig. 1E and F, the p32 mRNA and protein levels were equivalent during the infection with both attenuated and virulent strains, thus demonstrating that same amount of viruses have been used.

Taking together, these results reveal two important aspects regarding IFN-β production by attenuated versus virulent ASFV strains. First, the attenuated NH/P68 virus induces the production of IFN-β in infected macrophages, which in turn stimulates the expression of IFN-β-dependent genes such as IFIT-1 and CXCL10. Second, the virulent Armenia/07 strain is able to efficiently block IFN-β mRNA synthesis and production in infected macrophages.

### The STING pathway is blocked by virulent Armenia/07, while it is activated by attenuated NH/P68.

Given the results described above, we set out to dissect the molecular mechanism of IFN-β inhibition/induction by different ASFV strains in infected porcine alveolar macrophages.

It has been shown that induction of IFN-β synthesis depends mainly on the cGAS-STING pathway, which is triggered by the sensing of dsDNA in the cytoplasm (3). We therefore studied the correlation between IFN-β production and cGAS-STING pathway activation in macrophages after infection with attenuated or virulent ASFV.

**FIG 2:**
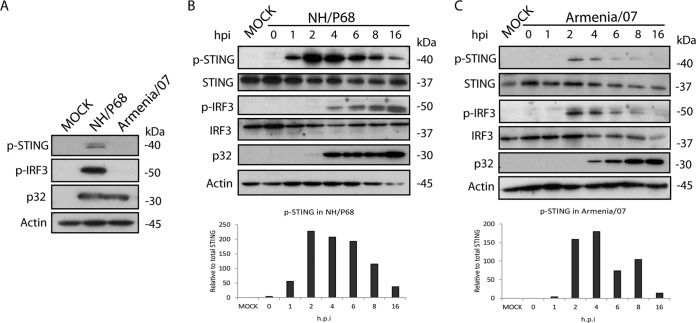
Effect of NH/P68 and Armenia/07 infection on STING and IRF3 phosphorylation. Porcine alveolar macrophages were mock infected or infected with NH/P68 or Armenia/07 strains (2 PFU/cell). Cells were lysed in RIPA buffer at 16 hpi (A) or at 0, 1, 2, 4, 6, 8, and 16 hpi with NH/P68 (B) or Armenia/07 (C), and lysates were separated by 7 to 20% SDS-PAGE, followed by immunoblotting with anti-p-STING, anti-STING, anti-p-IRF3, anti-IRF3, anti-ASFV p32 (viral early p32), and anti-actin antibodies. Graphs below the panels B and C indicate the relative phosphorylated-STING versus total STING protein. Densitometry was performed using ImageJ.

As seen in Fig. 2A, infection with NH/P68, but not with Armenia/07, induces phosphorylation of STING and IRF3 at 16 hpi, indicating that the STING pathway is activated in response to the infection with the attenuated, but not with the virulent, ASFV strain. STING phosphorylation occurs as early as 1 hpi, reaches a peak at 2 to 4 hpi and decreases by 16 hpi during NH/P68 infection (Fig. 2B). Meanwhile, IRF3 phosphorylation, which controls IFN-β transcription (2), starts at 4 hpi and increases until 16 hpi (Fig. 2B). These data indicate that STING activation occurs early after NH/P68 infection and before IRF3 phosphorylation and is maintained throughout 16 h of infection; this supports the IFN-β production data presented in Fig. 1.

In contrast, infection of macrophages with Armenia/07 produces only a weak phosphorylation signal of STING after 2 and 4 hpi, which decreases at later time points and is almost negative at 16 hpi (Fig. 2C). A similar activation profile was seen for IRF3 (Fig. 2C).

This demonstrates that—after initial moderate activation—virulent Armenia/07 is able to inhibit STING and IRF3 phosphorylation at later time points after infection; similar results were observed in regard to IFN-β synthesis (Fig. 1B).

### The specific inhibitor of cGAS, Ru521, inhibits the activation of STING mediated by NH/P68 and Armenia/07 infection.

cGAS is a cellular sensor for cytoplasmic dsDNA detection, which further induces STING pathway activation (27–29). To test the role of cGAS in sensing of ASFV dsDNA, porcine alveolar macrophages were mock treated or treated with increasing concentrations of the specific cGAS inhibitor Ru521 (30) for 30 min. Cells were then mock infected or infected with NH/P68 and Armenia/07 at a multiplicity of infection (MOI) of 2 for 5 h or 2 h, a time by which pSTING was shown to be maximum for attenuated and virulent strains, respectively (Fig. 2B and C). The cells were then collected, and the STING phosphorylation level was analyzed by Western blotting.

**FIG 3:**
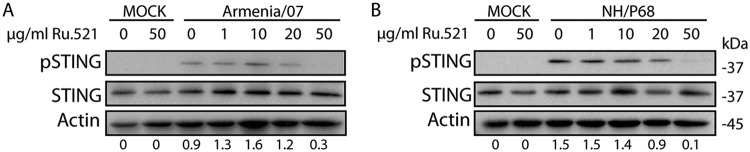
The activation of STING by NH/P68 and Armenia/07 is dependent on the cGAS sensor. Porcine alveolar macrophages were untreated or treated with 1, 10, 20, or 50 μg/ml of Ru521 and mock infected or infected with NH/P68 or Armenia/07 (2 PFU/cell). At 2 or 5 h after Armenia/07 or NH/P68 infection, respectively, the cells were lysed in RIPA buffer, and lysates were separated by 7 to 20% SDS-PAGE, followed by immunoblotting with anti-p-STING, anti-STING, or anti-actin antibodies as a control. Numbers below the bands indicate the relative level of phosphorylated STING. Densitometry was performed using ImageJ.

As Fig. 3 shows, STING phosphorylation was not detected in mock-infected cells, independent of Ru521 treatment. As expected by this time of infection, during both NH/P68 and Armenia/07 infections STING was phosphorylated in the nontreated cells; however, interestingly, the level of STING phosphorylation decreased when Ru521 at concentrations of 20 and 50 μg/ml was added at the beginning of the infection. These results indicate the involvement of cGAS in the activation of the STING pathway triggered, at early times after infection, either by NH/P68 or Armenia/07 ASFV strains.

### Infection of NH/P68 phosphorylates IRF3 and induces its binding to chromatin.

**FIG 4:**
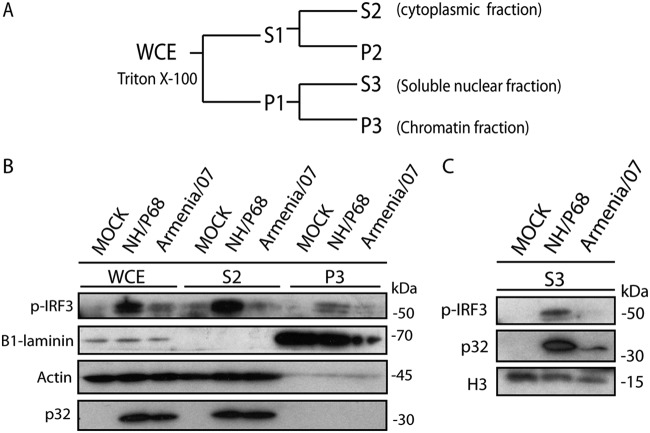
The activation of cGAS-STING by the attenuated strain NH/P68 promotes the translocation to the nucleus of p-IRF3. (A) Scheme of the biochemical fractionation method. See Materials and Methods for details. (B) Porcine alveolar macrophages were mock infected or infected with NH/P68 or Armenia/07 strain (2 PFU/cell). At 8 hpi the cells were collected, and chromatin fractionation was performed. The WCE, cytoplasmic fraction (S2), chromatin fraction (P3), and soluble nuclear fraction (S3) were separated by 7 to 20% SDS-PAGE, followed by immunoblotting with anti-p-IRF3, anti-β1-laminin, anti-H3 (histone 3), anti-ASFV p32 (viral early p32), and anti-actin antibodies.

As mentioned above, we have observed that infection with NH/P68 is able to substantially activate the cGAS-STING pathway. It has been described previously that the activation of STING and TBK1 induces the phosphorylation and then translocation of phosphorylated IRF3 (p-IRF3) to the nucleus (31, 32), where it acts as a transcription factor for IFN-β. In order to analyze the phosphorylation status and cellular localization of IRF3 during attenuated versus virulent ASFV infections, we used a specific antibody to detect p-IRF3 in different subcellular locations of the cell. To achieve this, mock-infected, NH/P68-infected, or Armenia/07-infected macrophages were fractionated after 8 hpi, a time point when both STING and IRF3 were found to be phosphorylated by NH/P68 but not by Armenia/07 (Fig. 2B and C). Cytoplasmic (S2) and nuclear (P1) fractions were obtained from the respective whole-cell extracts (WCE). Next, the P1 fraction was further separated into nuclear soluble fraction (S3) and chromatin fraction (P3) (Fig. 4A). We used specific markers for each cell fraction to confirm the accuracy of the fractionating procedure: actin for the soluble fraction, histone 3 (H3) for the soluble nuclear fraction, and β1-laminin for the chromatin fraction.

As shown in Fig. 4B and p-IRF3 is found in WCE from NH/P68 infected cells, whereas it was not found in mock-infected cells or only in small amounts in Armenia/07-infected cells. After cell fractionation, p-IRF3 is mainly detected in the cytoplasmic fraction of alveolar macrophages infected with NH/P68, as well as in the soluble nuclear fraction (S3) (Fig. 4C). p-IRF3 was also detected in the chromatin fraction (P3) of NH/P68-infected cells, suggesting that at 8 h after NH/P68 infection, functional p-IRF3 binds to chromatin, possibly acting as transcription factor for IFN-β.

Only weak or no bands corresponding to p-IRF3 were detected either in the chromatin fraction (P3) or in the nuclear soluble fraction (S3) in uninfected cells or in cells infected with Armenia/07. These data suggest that the cGAS-STING pathway is strongly activated during NH/P68 infection, resulting in the activation and translocation of p-IRF3 to the nuclear chromatin fraction, where it activates synthesis of IFN-β. In contrast, ASFV Armenia/07 displays mechanisms which almost completely inhibit p-IRF3 activation and translocation to the nucleus; this resulted in weak or no induction of IFN-β synthesis.

### Armenia/07-induced blockage of the cGAS-STING pathway happens early during viral infection.

**FIG 5:**
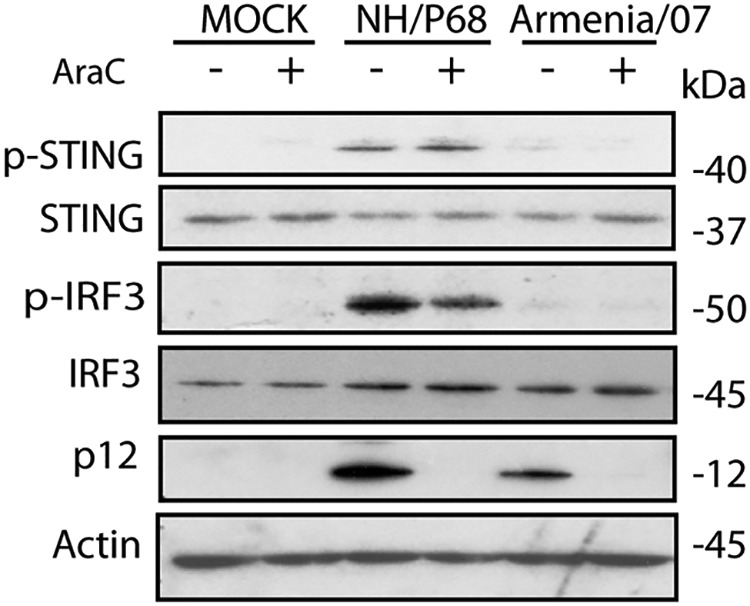
The blocking of the cGAS-STING pathway mediated by Armenia/07 depends on the products of viral early gene(s). Porcine alveolar macrophages were mock infected or infected with NH/P68 or Armenia/07 strain (2 PFU/cell) and untreated or treated with 40 μg/ml of cytosine arabinoside (AraC). At 16 h postinfection, the cells were lysed in RIPA buffer, and lysates were separated by 7 to 20% SDS-PAGE, followed by immunoblotting with anti-p-STING, anti-STING, anti-p-IRF3, anti-IRF3, anti-1262, and anti-actin antibodies, respectively.

ASFV displays a very accurate temporal kinetics of replication in the infected cell, producing early-early, early-late, and late viral mRNAs. These differently timed messenger RNAs are the products of viral genes whose transcription is regulated before or after viral DNA replication (8). In order to determine at what step of the Armenia/07 replication the cGAS-STING route is blocked, we used cytosine arabinoside (AraC), an inhibitor of viral DNA replication, widely used to prevent the expression of late ASFV genes (8). In order to determine whether the cGAS-STING inhibition produced by Armenia/07 occurs at early or late times of ASFV replication, we infected alveolar macrophages in the presence or absence of AraC and analyzed the phosphorylation of both STING and IRF3. The results showed that the cGAS-STING pathway inhibition by Armenia/07 occurs at a time point prior to the replication of viral DNA. No phosphorylation of STING and IRF3 was observed in the presence of AraC (Fig. 5), indicating that early viral genes and/or gene products are involved in this inhibition exerted by the virulent ASFV strain.

As a control for both the infection and the effects of AraC on viral DNA replication, we employed a specific antibody to assess the expression of the viral protein p12, a late viral product, which should be only expressed in the absence of AraC. As shown in the Fig. 5, p12 is only found in the lanes corresponding to infected cells in the absence of AraC.

In contrast, NH/P68 infection induces activation of the cGAS-STING route in the presence and absence of AraC, since phosphorylation of both STING and IRF3 are observed. This indicates that the replication of NH/P68 viral DNA is not necessary to activate the cGAS-STING pathway.

### Armenia/07 blocks STING and TBK1 phosphorylation by a cGAMP-dependent mechanism.

**FIG 6:**
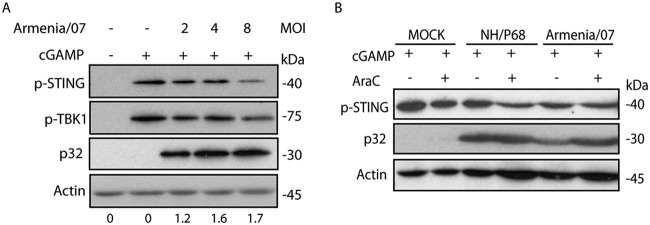
Armenia/07 blocks the phosphorylation of STING and TBK1 mediated by cGAMP. (A) Porcine alveolar macrophages were mock infected or infected with Armenia/07 strain with 2, 4, or 8 PFU/cell. At 5 hpi, the cells were untreated or treated with 10 μg/ml of cGAMP. At 2 h after cGAMP treatment, the cells were collected. Numbers below the bands indicate the relative levels of p32 viral protein. Densitometry was performed using ImageJ. (B) Porcine alveolar macrophages were mock infected or infected with NH/P68 or Armenia/07 strain (2 PFU/cell) and untreated or treated with 40 μg/ml of cytosine arabinoside (AraC). At 14 hpi, the cells were untreated or treated with 10 μg/ml of cGAMP. At 2 h after cGAMP treatment, the cells were collected. (A and B) Cells were lysed in RIPA buffer, and lysates were separated by 7 to 20% SDS-PAGE, followed by immunoblotting with anti-p-STING, anti-p-TBK1, anti-ASFV p32 (viral early p32 protein), and anti-actin antibodies.

Immediately after sensing cytoplasmic dsDNA, cGAS is activated to synthesize cGAMP from ATP/GTP; cGAMP binds STING, inducing its phosphorylation, dimerization, and activation (2). In order to analyze the mechanism that Armenia/07 uses to block STING phosphorylation and to determine whether cGAMP is involved, we infected macrophages with increasing MOIs of Armenia/07 (Fig. 6A). At 5 hpi, a time point by which Armenia/07 fully inhibits STING phosphorylation (see Fig. 2C), cGAMP (10 μg/ml) was added to the cultures. After an two additional hours of incubation (7 hpi), the phosphorylation of both STING and TBK1 was analyzed by Western blotting.

As shown in Fig. 6A, treatment of the noninfected cells with cGAMP induces phosphorylation of STING and TBK1. In the presence of cGAMP, a decrease in the amounts of both p-STING and p-TBK1 was observed when cells were infected with Armenia/07 at an MOI of 8; this indicates that the virulent virus is able to reverse the STING and TBK1 activation induced by cGAMP in a dose-dependent manner, probably also involving a c-GAS-dependent mechanism. This result also shows that the viral process to inhibit STING/TBK1 activation can be overcome by cGAMP, suggesting that cGAMP is a critical component of the activation pathway. A specific monoclonal antibody against the ASFV protein p32 was used as a control for ASFV infection of the cells.

In an additional experiment, cGAMP (10 μg/ml) was added after 14 h of infection with the attenuated or the virulent ASFV strains and then incubated for two additional hours with or without AraC (Fig. 6B). Exogenous cGAMP caused the phosphorylation of STING in noninfected cells, which is also observed during NH/P68 infection. Interestingly, the inhibition of STING phosphorylation induced by Armenia/07 (Fig. 2C and 5) was reversed by exogenous cGAMP, confirming that Armenia/07 inhibits STING phosphorylation by a mechanism that involves cGAMP. To assess whether these events are dependent on viral DNA replication, cells were treated with 40 μg/ml of AraC. There was no difference whether the cells were treated or not with AraC, suggesting that both the STING blockage by Armenia/07 and the rescue by cGAMP are mechanisms independent of viral replication, suggesting in turn that very early or early viral factors are involved.

### STING localizes at perinuclear microsomes in cells infected with NH/P68 but not in cells infected with Armenia/07.

As mentioned above, Armenia/07 blocks the cGAS-STING pathway by using early viral effectors downstream of cGAS activation. STING activation requires posttranslational modifications, dimerization, and transport from the ER through the Golgi to perinuclear structures, which are critical for its function (33).

**FIG 7:**
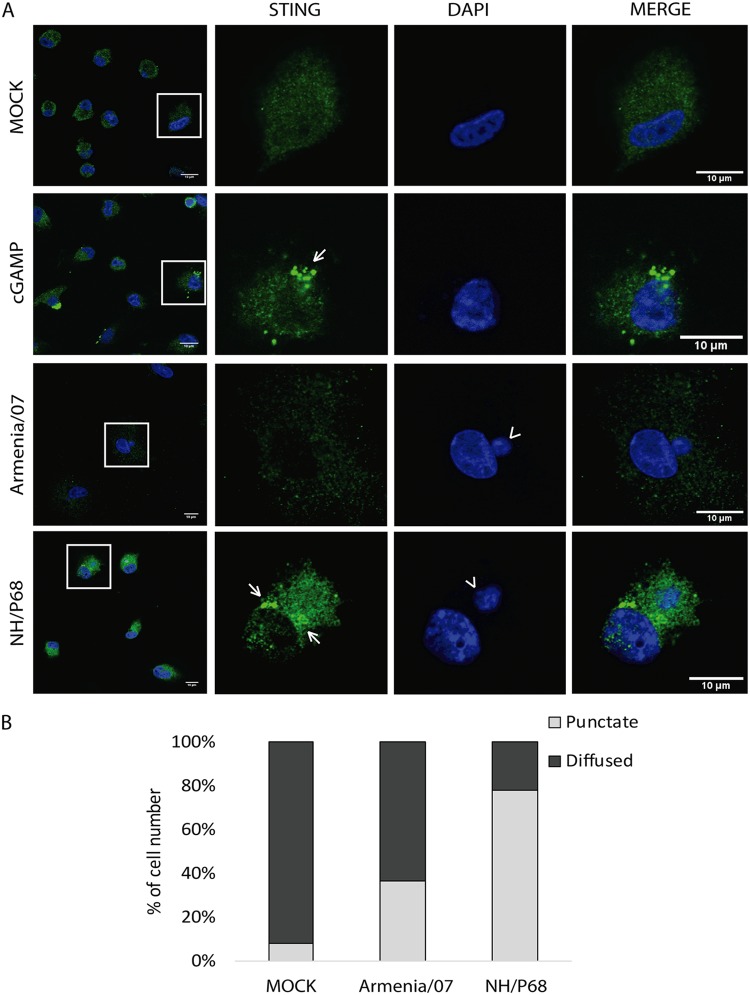
STING locates at perinuclear clusters in porcine alveolar macrophages during NH/P68 but not during Armenia/07 infection. (A) Porcine alveolar macrophages were mock infected or infected with NH/P68 or Armenia/07 (5 PFU/cell) or treated with cGAMP (20 μg/ml). At 6 hpi or 1 h after cGAMP treatment, the cells were fixed and stained with DAPI and anti-STING antibody and then examined by using a confocal microscope. Arrows indicate punctuated perinuclear structures. An arrowhead indicates viral factories. (B) Quantitative analysis of cells with punctate pattern or diffused signal is presented. The percentage of cell number was calculated among 75 cells under each condition.

To investigate the localization of STING during ASFV infection, porcine alveolar macrophages were mock infected or infected at an MOI of 2 for 6 h with Armenia/07 or NH/P68. cGAMP-treated macrophages were used as a positive control of STING activation (20 μg/ml), and the samples were then processed for immunofluorescence by using a specific anti-STING antibody. As observed in Fig. 7A, cGAMP-treated cells and cells infected with NH/P68 show STING localization in punctuate perinuclear structures (or microsomes; highlighted by arrows). STING localization in microsomes indicates its activation and has been described as necessary for further activation of downstream components of the STING pathway (34). In contrast, cells infected with Armenia/07 present a more diffuse STING distribution pattern throughout the cytoplasm, similar to that found in mock-infected cells. The latter results indicate a nonactivation status of STING and confirms the small amount of p-STING we found during Armenia/07 infection (Fig. 2C). Both Armenia/07 and NH/P68 infections were confirmed by the presence of viral factories, which are stained by DAPI (4′,6′-diamidino-2-phenylindole) as small DNA accumulations next to the cell nucleus (arrowheads). We quantified STING distribution pattern in infected cells, which were identified by the presence of viral factories. This analysis revealed that in more than 80% of NH/P68-infected cells STING is localized in punctuated perinuclear microsome structures (Fig. 7B). In contrast, STING was localized in microsomes in less than 40% of Armenia/07-infected cells (Fig. 7B).

Taken together, these results indicate that Armenia/07 induces a blockage in the activation and intracellular transport of STING, whereas NH/P68 infection does not, resulting in a typical punctuate pattern surrounding the nucleus characteristic of STING activation.

### Differences in STING trafficking during NH/P68 versus Armenia/07 infection.

STING undergoes posttranslational modifications necessary for its activation during its traffic through the Golgi network. In particular, during the passage through the *trans*-Golgi network (TGN), activated STING induces TBK1 activation, which is a key component in the pathway to activate IFN-β production (34). Taking into account the inhibition found on STING trafficking mediated by virulent Armenia/07 (Fig. 7), we further assessed the impact that virulent strain exerts on the cellular transit of STING.

**FIG 8:**
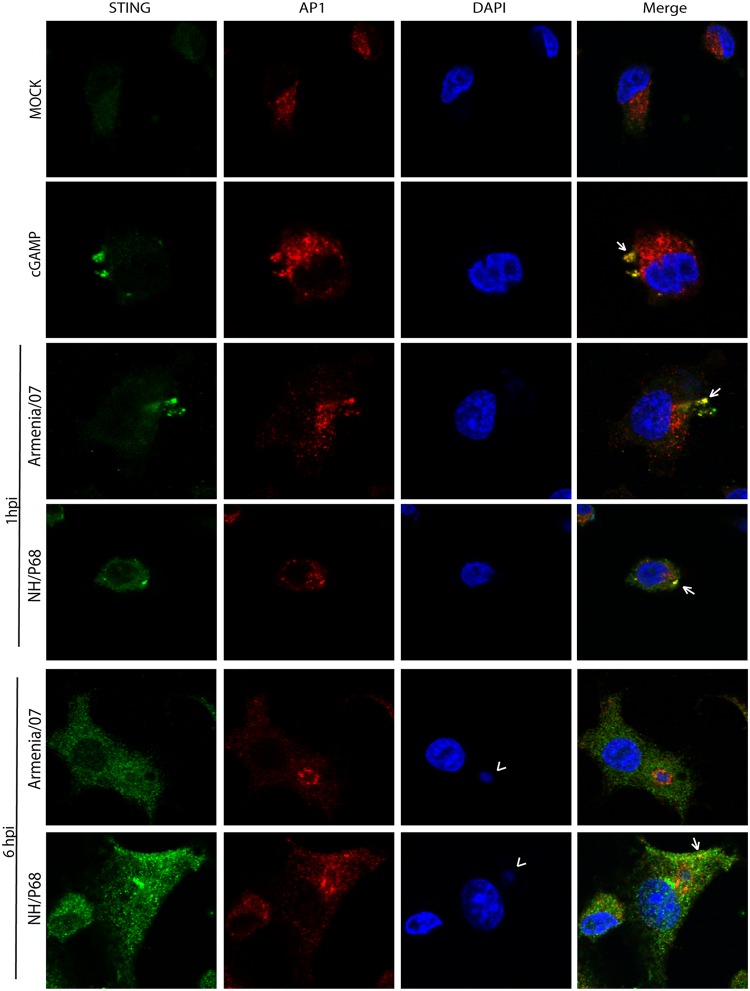
Differences on STING movement through the TGN during very early or early NH/P68 and Armenia/07 infections. Porcine alveolar macrophages were mock infected or infected with NH/P68 or Armenia/07 strain (2 PFU/cell) or treated with cGAMP (20 μg/ml). At 1 and 6 hpi or 1 h after cGAMP-treatment, the cells were fixed and stained with DAPI, anti-STING, and anti-AP1 antibodies and then examined by using a confocal microscope. An arrowhead indicates viral factories. Arrows indicate colocalization between STING accumulations and AP1.

We hypothesized that the activation of STING, and therefore its traffic through the TGN, should be an event occurring at early times after infection, since we have found STING phosphorylation in the presence of AraC. Thus, we compared STING distribution either at “very early” (1 hpi) or at “early” (6 hpi) times after infection with attenuated or virulent ASFV strains. To do this, we used either NH/P68 or Armenia/07 at an MOI of 2 to infect porcine alveolar macrophages, together with cGAMP-treated macrophages as a positive control of STING activation; the cells were then processed for immunofluorescence, and specific antibodies against STING and against the AP1 complex (marker for TGN [16, 35]) were applied. As shown in Fig. 8 (central panels), STING colocalizes with AP1 (arrows) in both NH/P68- and Armenia/07-infected cells after 1 h of infection. A similar colocalization pattern between STING and AP1 was found in cGAMP-treated macrophages (Fig. 8). This indicates that both attenuated and virulent ASFV strains “activate” STING trafficking very early after infection. By 6 hpi, STING accumulates in punctuated perinuclear structures in cells infected with NH/P68 but not with Armenia/07 (see Fig. 8, lower panels). Furthermore, STING partially colocalizes with AP1 in cytoplasmic structures outside from perinuclear structures in NH/P68-infected cells. Importantly, this colocalization pattern was not detected in cells infected with Armenia/07 (Fig. 8, lower panels). AP1 is located mainly around the ASFV viral factories (arrowhead) in both Armenia/07- and NH/P68-infected cells, a localization that was previously described (16).

**FIG 9:**
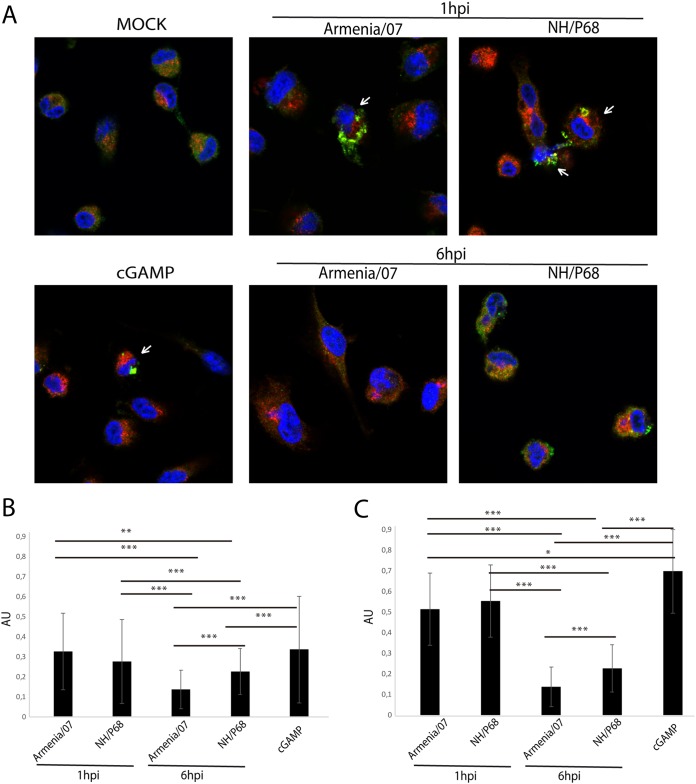
Quantification of STING colocalization with AP1 at very early or early time points after NH/P68 and Armenia/07 infections. (A) Porcine alveolar macrophages were mock infected or infected with NH/P68 or Armenia/07 strain (2 PFU/cell) or treated with cGAMP (20 μg/ml). At 1 and 6 hpi or 1 h after cGAMP treatment, the cells were fixed and stained with DAPI (blue), anti-STING (green), and anti-AP1 (red) antibodies and examined by using a confocal microscope. Cells where STING exhibit a punctuate pattern are highlighted (arrows). (B and C) Quantification of fraction of STING overlapping AP1 (Mander’s coefficient) for each condition (B) and only for cells with a STING punctuated pattern (arrows) at 1 hpi and cGAMP-treated cells (C). The data are means ± the SD (*n* = 50). Data were statistically analyzed by using a Student *t* test (*, *P* < 0.05; **, *P* < 0.01; ***, *P* < 0.001).

The degree of colocalization pattern observed under these conditions (Fig. 9A) was quantified to determine the fraction of STING overlapping AP1 (Mander’s coefficient). As shown in Fig. 9B, the highest colocalization between STING and AP1 was found in both NH/P68- and Armenia/07-infected cells after 1 h of infection, similarly to cGAMP-treated cells. This colocalization degree strongly increases if quantification is restricted to cells where STING is activated, forming punctuate structures (Fig. 9A, see arrows, and Fig. 9C). These data emphasize that STING activates and traffics through the TGN during both Armenia/07 and NH/P68 infection at the very early time of 1 hpi. However, the STING-AP1 colocalization significantly decreased at 6 hpi and more specifically in Armenia/07- versus NH/P68-infected cells, as shown in Fig. 9B and C.

Altogether, these results suggest that whereas both virulent and attenuated ASFV strains trigger the cGAS-STING pathway at very early times postinfection, Armenia/07 is able to inhibit STING activation during later times of infection, thus impairing its activation and traffic to perinuclear microsomes.

## DISCUSSION

The manner how two different ASFV strains ranging in virulence affect IFN-β production of infected cells through the control of the cGAS-STING pathway has been addressed in detail in the present study. One ASFV strain is NH/P68, a nonfatal, naturally occurring, nonhaemadsorbing virus, isolated from a chronically infected pig (36), and the other ASFV strain is Armenia/07, a highly virulent virus currently circulating in Europe, Russia, and China (37). Our results demonstrate that whereas Armenia/07 inhibits the synthesis of IFN-β (and its dependent genes IFIT-1 and CXCL10) by inhibiting IRF3 activation and STING phosphorylation, NH/P68 does not. Infection of porcine alveolar macrophages with NH/P68 results in (i) the induction of significant levels of IFN-β, (ii) phosphorylation of STING, (iii) traffic of STING through the Golgi to perinuclear punctuated structures, and (iv) localization of IRF3 to the nuclear soluble fraction and binding to chromatin. Furthermore, inhibition of cGAS by the specific inhibitor Ru521 (30) impairs NH/P68-induced STING phosphorylation, suggesting that cGAS is the main DNA sensor activating the STING cascade by the attenuated ASFV NH/P68 isolate. In contrast, cGAMP-induced phosphorylation of STING was prevented by infection of cells with Armenia/07. Also, STING trafficking to perinuclear punctuated structures was severely impaired in Armenia/07-infected cells, indicating that the virulent ASFV strain prevents the activation and trafficking of STING.

Differences in the IFN-β mRNA expression levels in infected cells have been reported for other virulent and attenuated ASFV strains (38). Similar observations were made in previous reports describing the inhibition of IFN-α production during virulent ASFV strain infection (39, 40). In contrast, Portugal et al. reported recently that IFN-α inhibition was exerted by both virulent and attenuated ASFV strains (41). Similarly, when IFN-α and IFN-β were analyzed in sera from animals infected with virulent ASFV strains (42, 43), induction of IFN-α and IFN-β was observed, which was attributed to cytokine production by non-ASFV-susceptible cells. Since there are most likely several pathways and virulence factors involved in ASFV virulence, this result is not surprising.

It is noteworthy that whereas our data show a strong inhibition of IFIT-1 mRNA expression during Armenia/07 infection, in comparison with the expression during NH/P68 infection, the amount of IFN-β secreted after 8 hpi with Armenia/07 does not affect IFN-β-dependent gene IFIT-1 expression. One possible explanation would be that virulent strain may encode genes functionally homologous to type I IFN receptors as has been described for vaccinia and other poxvirus (44), in such a way that the produced IFN is “neutralized” at this time point of the virulent infection (8 hpi), impairing the activation of IFN-dependent genes.

The cGAS-STING pathway, which leads to the production of IFN-β, is critical for the innate immune response and is therefore targeted by several viruses (45). Here, we show for the first time that IFN-β production during ASFV infection is controlled via the cGAS-STING pathway. Importantly, early after infection the cGAS-STING pathway was induced by both attenuated and virulent ASFV, but later on, was effectively inhibited only by virulent Armenia/07. Our data reveal a clear correlation between ASFV virulence and virus ability to inhibit the cGAS-STING pathway. Similar results have been obtained with vaccinia virus, an ASFV-related, dsDNA cytoplasmic virus. The attenuated modified vaccinia virus Ankara (MVA) induces IFN-β in infected cells via the activation of the cGAS-STING pathway (46), while virulent vaccinia strains prevent cGAS-STING activation and hence IFN-β production in infected cells (47). Our results, therefore, reinforce the idea that ASFV virulence versus attenuation, two concepts controversially discussed in the ASFV field, may be phenomena associated with the control of the cGAS-STING pathway.

Activation of the cGAS-STING pathway involves the phosphorylation and translocation of STING from the ER through the Golgi compartment, the phosphorylation of both TBK1 and IRF3, and the subsequent translocation of p-IRF3 to the nucleus. Using the viral DNA replication inhibitor AraC, we showed that STING phosphorylation is impaired by Armenia/07 in the presence of AraC, thus involving events happening before viral DNA replication. In contrast, STING phosphorylation is triggered as early as 1 h after NH/P68 infection and leads to the phosphorylation of IRF3, a transcription factor regulating IFN-β synthesis (31). The different time points at which IRF3 is phosphorylated during attenuated versus virulent strains suggest that a faster transition between activated STING and phosphorylated IRF3 occurs during virulent strain infection. Phosphorylated IRF3 then translocates to the nucleus and binds to the chromatin fraction to induce IFN-β production. The latter happens during NH/P68 infection, whereas during Armenia/07 infection, IRF3 was not phosphorylated and consequently absent from the nuclear and chromatin fractions of the infected cells. Interestingly, it has been recently reported (48) that STING phosphorylation is required to further activate IRF3.

Similarly, STING activation and p-IRF3 have been found in the nuclear fraction in cells infected with influenza virus (49) and the gammaherpesvirus rhesus rhadinovirus (50), which are both able to induce IFN-β. Furthermore, during MVA versus virulent poxvirus WR infection, an opposing regulation of the IFN-β has been reported. The authors showed that attenuated MVA virus induced activation of the cGAS-STING pathway, whereas inhibition of this pathway was observed during virulent WR infection (46, 47); this behavior is similar to what we report here in relation to the regulation of the cGAS-STING pathway by NH/P68 and Armenia/07 in infected cells.

STING is a prototype downstream effector, which can be modulated by several viral products in order to counteract the cGAS-STING pathway and type I IFN production (51–57). Activation of STING during viral infection can occur through the triggering of different cytoplasmic dsDNA sensors (58–60). cGAS was identified as the main sensor of this pathway in both humans and mice (27–29), playing an important role in type I IFN response against DNA viruses, including HSV-1, KSHV, and vaccinia virus (2, 46, 56). Importantly, we have been able to identify cGAS as the main DNA sensor involved in STING activation upon ASFV infection in porcine alveolar macrophages. In fact, we found that a specific inhibitor of cGAS, Ru521 (30), blocked the activation of STING produced by NH/P68 infection. However, we cannot exclude the involvement of other sensors such as IFI16 or sensors triggered in the absence of active cGAS (61, 62).

In addition, we showed that cGAMP, the cGAS-activated product mediating the activation of STING, was counteracted during Armenia/07 but not NH/P68 infection, suggesting that the viral mechanism displayed by Armenia/07 to inhibit STING activation somehow competes with cGAMP. It has been described that various viruses can act at different levels to counteract the IFN-β activation pathway (45). Here, we show that virulent Armenia/07 is able to counteract cGAMP-dependent STING activation, even in the presence of exogenous cGAMP. However, alternative inhibition modes exerted by Armenia/07 or by other virulent ASFV strains cannot be excluded, and future experiments are planned to assess this interesting possibility.

It is remarkable that the activation of STING, after phosphorylation and dimerization, involves trafficking from the ER through the Golgi compartment to perinuclear punctuated structures, called microsomes (33, 34, 63). Significantly, STING appears in these microsomes in most of the NH/P68-infected cells at 6 hpi, whereas at that time postinfection with Armenia/07, STING is mainly found throughout the cytoplasm. Transit of STING through the Golgi, prior to its translocation into microsomes, has been shown to be a key event for STING activation, since STING undergoes posttranslational modifications and interaction with molecules such as TBK1 (34, 64). Therefore, to further assess the virulence mechanism of ASFV, we investigated whether the transit of STING through the Golgi is blocked during Armenia/07 infection, as reported for other viruses such as HSV-1 or human cytomegalovirus (57, 65). At very early times after both NH/P68 and Armenia/07 infection, STING traffics through the TGN in a similar manner than in cGAMP-treated cells, while at 6 hpi this traffic is prevented in Armenia/07-infected cells but not in NH/P68-infected cells, thus impairing the ability of STING to reach microsomes. These results suggest that at very early times of infection, STING is activated in both NH/P68 and in Armenia/07 infected cells, but several hours later, the virulent ASFV strain is able to block activation of STING.

The involvement of the cGAS-STING pathway in the control of IFN-β production during ASFV infection has not been described so far, and therefore the potential viral candidate genes and/or the respective molecular mechanisms involved in blocking of the cGAS-STING pathway are not yet known. From the results presented here, possible viral gene candidates could be very early genes, such as products of the so-called multigene families (66). In this regard, very recently, studies in human embryonic kidney (HEK) cells, have postulated that the transfected DP96R gene of the Chinese virulent strain 2018/1 could have a role in the control of the cGAS-STING pathway (24). However, these results should be discussed cautiously, since (i) HEK cells are not susceptible to ASFV infection and (ii) these studies have not been performed in the context of an active ASFV infection. In addition, the DP96R gene is present and identical in both attenuated and virulent strains of the same genotype, such as NH/P68 and Ba71 (both genotype I) and has 98% identity between attenuated and virulent strains of different genotypes, such as NH/P68 (genotype I) and Armenia/07 (genotype II). Therefore, the experiments performed above with DP96R most likely do not explain the differences in the cGAS-STING pathway modulation exerted by virulent and attenuated ASFV strains.

Future *in vitro* and *in vivo* studies are necessary to elucidate the molecular mechanisms of control of the cGAS-STING pathway by virulent strains of ASFV.

## MATERIALS AND METHODS

### Cell culture and virus infections.

Porcine alveolar macrophages (PAM) were prepared by bronchoalveolar lavage as described previously (67) and grown in Dulbecco modified Eagle medium (DMEM) supplemented with 2 mM l-glutamine, 100 U/ml gentamicin, nonessential amino acids, and 10% porcine serum. Cells were grown at 37°C in a 7% CO_2_ atmosphere saturated with water vapor. COS-1 cells (from African green monkey kidney) were obtained from the American Type Culture Collection (ATCC) and grown in DMEM supplemented with 2 mM l-glutamine, 100 U/ml gentamicin, nonessential amino acids, and 2 to 5% fetal bovine serum (Invitrogen Life Technologies). The ASFV isolates Armenia/07 and NH/P68 were propagated on PAM. Briefly, subconfluent PAM cells were cultivated in p150 plates and infected with ASFV at an MOI of 0.2 PFU/cell in DMEM–10% porcine serum. At 96 hpi, the cells were recovered and centrifuged at 3,000 rpm for 15 min. The cell pellet was discarded. The supernatant containing the viruses was clarified at 14,000 rpm for 6 h at 4°C, resuspended in medium, and stored at −80°C. The viruses were then titrated by plaque assay on COS-1 cells as previously described (64). Infection was performed after ASFV adsorption at 37°C for 90 min, when the inoculum was removed, and fresh medium was added. The cells were then incubated at 37°C for the times indicated (in hpi).

### Antibodies and reagents.

Polyclonal rabbit anti phospho-STING antibody (Ser366, catalog no. 85735), monoclonal rabbit anti phospho-IRF3 antibody (Ser396, catalog no. 4947), and monoclonal rabbit anti phospho-TBK1/NAK antibody (Ser172, catalog no. 5483) were purchased from Cell Signaling. Monoclonal mouse anti-IRF3 antibody (SL-12, sc-33641), anti-m-IgGκ secondary antibody (BP-HRP, sc-516102), polyclonal goat anti actin antibody (I-19, sc-1616), and polyclonal goat anti β-laminin (S-20) were purchased from Santa Cruz Biotechnology. Polyclonal rabbit anti TMEM173/STING antibody (19851-1-AP) was purchased from Proteintech. Polyclonal rabbit anti-histone H3 antibody (ab18521) was acquired from Abcam. Antiserum 1262 was kindly provided by E. Tabarés; monoclonal mouse anti-p32 (S-1D8) was kindly provided by S.-Y. Sunwoo. Anti-rabbit/Alexa Fluor 488 and anti-mouse/Alexa Fluor 555 antibodies were purchased from Invitrogen. Anti-rabbit and anti-mouse immunoglobulin G coupled to peroxidase and ECL Prime Western blotting detection reagent were acquired from Amersham Biosciences. Monoclonal mouse anti-γ-adaptin antibody (AP-1, A4200), anti-goat antibody G coupled to peroxidase, and digitonin were acquired from Sigma-Aldrich.

### Cellular fractionation.

PAM were seeded in p60 plates (6 × 10^6^ cells/plate) and mock or ASFV infected (2 PFU/cell) for 8 h. The WCE, cytoplasmic fraction (S2), soluble nuclear fraction (S3), and chromatin fraction (P3) were isolated as described previously (68). Briefly, cells were resuspended in buffer A (10 mM HEPES [pH 7.9], 10 mM KCl, 1.5 mM MgCl_2_, 0.34 M sucrose, 10% glycerol, 1 mM dithiothreitol [DTT], 0.1mM phenylmethylsulfonyl fluoride [PMSF], and protease and phosphatase inhibitors [Roche]) and Triton X-100 were added (separating a part for WCE), followed by incubation for 5 min on ice. The nuclei were then centrifuged at 3,600 rpm 4 min at 4°C. The first supernatant (S1) was centrifuged at 14,000 rpm 15 min at 4°C, and the second supernatant was stored (S2). The first pellet (P1) was washed with buffer A and later was resuspended in buffer B (3 mM EDTA, 0.2 mM EGTA, 1 mM DTT, 0.1 mM PMSF, and protease and phosphatase inhibitors [Roche]), incubated 30 min on ice, and centrifuged at 4,000 rpm for 4 min at 4°C. The third supernatant (S3) was stored, and the third pellet (P3) was washed with buffer B and finally resuspended in loading buffer.

### cGAMP and Ru521 treatment.

Cells were treated with a specific concentration of 2′3′-cGAMP (Invivogen) or cGAS inhibitor Ru521 (Invivogen) in digitonin permeabilization buffer (50 mM HEPES [pH 7.0], 100 mM KCl, 85 mM sucrose, 3 mM MgCl_2_, 0.2% bovine serum albumin [BSA], 1 mM ATP, 0.1 mM DTT, and 10 μg/ml digitonin) (28). The cells were incubated for 30 min at 37°C, and then the cells were washed with phosphate-buffered saline (PBS), and fresh medium or virus inoculation was added as indicated. For the Ru521-treated samples, cells were incubated for 2 h with fresh DMEM before being infected.

### ELISA.

PAM were seeded in 24-well plates (2 × 10^6^ cells/well) and mock or ASFV infected (4 PFU/cell) in DMEM–10% porcine serum. ASFV viral adsorption to cells was performed at 37°C for 90 min. The inoculum was then removed, and 250 μl of fresh DMEM medium without porcine serum was added. At the indicated times postinfection, cell culture supernatants were collected and assayed for porcine IFN-β using a porcine IFN-β ELISA kit (MyBioSource), as described by the manufacturer.

### Immunofluorescence.

PAM were grown on coverslips and treated or untreated with cGAMP (20 μg/ml) or mock or ASFV infected at the indicated MOI. At the indicated times postinfection (1 or 6 hpi) or 1 h after cGAMP treatment, the cells were fixed with 4% paraformaldehyde for 20 min at room temperature, permeabilized with 0.2% Triton X-100 for 15 min, and blocked with PBS–5% BSA for 45 min. The cells were stained with the primary antibodies anti-STING antibody (1/50) and anti-γ-adaptin (AP1) antibody (1/100) diluted in PBS–1% BSA for 1 h. The cells were then washed with PBS and incubated with the fluorescence-conjugated secondary antibodies anti-rabbit/Alexa Fluor 488 (1/500) and anti-mouse/Alexa Fluor 555 (1/500) diluted in PBS–1% BSA for 1 h. After a wash with PBS, the coverslips were mounted with DAPI Fluoromount-G (SouthernBiotech). Images were acquired by using a CLSM LSM710 coupled to an inverted AxioObserver microscope (Zeiss) with a 63× oil immersion objective lens and analyzed using ImageJ. Colocalization between STING and AP1 was quantified by calculating Mander’s coefficient (M1) using the plugin JACoP (Just Another Colocalization Plugin; Fiji).

### RT-qPCR assay.

PAM were seeded in p60 plates (6 × 10^6^ cells/plate) and mock or ASFV infected with 4 PFU/cell in DMEM–10% porcine serum. At 4, 8, or 16 hpi, the total RNA was harvested from cells using an RNeasy kit (Qiagen). cDNA was synthesized using a NZY first-strand cDNA synthesis kit (NZYTech). qPCR was performed using an CFX384 Touch real-time PCR detection system (Bio-Rad) with SYBR green master mix (Promega). Gene expression levels were normalized to the housekeeping gene (18S rRNA), and these values were then normalized to the mock-treated sample. The primers used were 5′-GGCCCGAGGTTATCTAGAGTC-3′ and 5′-TCAAAACCAACCCGGTCA-3′ for porcine 18S rRNA detection, 5′-TCAGGGCAAAGAGAGCCTTA-3′ and 5′-GGCCATTTTGTCTGAATGCT-3′ for porcine IFIT-1 detection, 5′-CAATGAAAAAGAATGGGGAGA-3′ and 5′-CCTTTCTTTGCTAATTGCTTTCA-3′ for porcine CXCL10 detection, 5′-AAAAATGATAATGAAACCAATGAATG-3′ and 5′-ATGAGGGCTCTTGCTCAAAC-3′ for viral p32 detection, and 5′-GTGGAACTTGATGGGCAGAT-3′ and 5′-TTCCTCCTCCATGATTTCCTC-3′ for porcine IFN-β detection.

### Western blot analysis.

PAM were cultivated as indicated and mock or ASFV infected. At the specified times postinfection, the cells were collected, washed with PBS, and lysed with radioimmunoprecipitation assay (RIPA) buffer supplemented with protease and phosphatase inhibitors (Roche) for 30 min at 4°C, sonicated, and centrifuged at 13,000 rpm for 10 min at 4°C. The supernatants were collected, and equal amounts of protein per sample were used. Samples were resolved by sodium dodecyl sulfate polyacrylamide gel electrophoresis (SDS-PAGE) and transferred to Immobilon-P membranes (Millipore). The membranes were incubated with the following specific primary antibodies: anti-STING antibody (1/1,000), anti-p-STING antibody (1/1,000), anti-IRF3 antibody (1/200), anti-p-IRF3 antibody (1/1,000), anti-p-TBK1 antibody (1/1,000), anti-actin antibody (1/5,000), anti-β-laminin antibody (1/1,000), anti-histone 3 antibody (1/2,000), anti-p32 antibody (1/5,000), and anti-1262 antibody (1/1,000) diluted in Tris-buffered saline (TBS) supplemented with 1% milk. Membranes were washed three times with TBS and exposed 1 h to specific peroxidase-conjugated secondary antibodies: anti-rabbit and anti-mouse immunoglobulin G coupled to peroxidase (1/5,000 and 1/2,000, respectively) from Amersham Biosciences and anti-m-IgGκ secondary antibody (1/1,000) from Santa Cruz Biotechnology. Chemiluminescence detection was performed using ECL Prime (Amersham Biosciences).
